# Binge-like Prenatal Ethanol Exposure Causes Impaired Cellular Differentiation in the Embryonic Forebrain and Synaptic and Behavioral Defects in Adult Mice

**DOI:** 10.3390/brainsci12060793

**Published:** 2022-06-17

**Authors:** Shivakumar Subbanna, Balapal S. Basavarajappa

**Affiliations:** 1 Center for Dementia Research, Nathan Kline Institute for Psychiatric Research, Orangeburg, NY 10962, USA; subbanna.shivakumar@nki.rfmh.org; 2 Molecular Imaging and Neuropathology Area, New York State Psychiatric Institute, New York, NY 10032, USA; 3 Department of Psychiatry, Columbia University Irving Medical Center, New York, NY 10032, USA; 4 Department of Psychiatry, New York University Langone Medical Center, New York, NY 10016, USA

**Keywords:** FASD, alcohol, development, brain, electrophysiology, cognition, psychiatric disorders, disabilities, synaptic plasticity, gestation, obsessive-compulsive disorder

## Abstract

An embryo’s in-utero exposure to ethanol due to a mother’s alcohol drinking results in a range of deficits in the child that are collectively termed fetal alcohol spectrum disorders (FASDs). Prenatal ethanol exposure is one of the leading causes of preventable intellectual disability. Its neurobehavioral underpinnings warrant systematic research. We investigated the immediate effects on embryos of acute prenatal ethanol exposure during gestational days (GDs) and the influence of such exposure on persistent neurobehavioral deficits in adult offspring. We administered pregnant C57BL/6J mice with ethanol (1.75 g/kg) (GDE) or saline (GDS) intraperitoneally (i.p.) at 0 h and again at 2 h intervals on GD 8 and GD 12. Subsequently, we assessed apoptosis, differentiation, and signaling events in embryo forebrains (E13.5; GD13.5). Long-lasting effects of GDE were evaluated via a behavioral test battery. We also determined the long-term potentiation and synaptic plasticity-related protein expression in adult hippocampal tissue. GDE caused apoptosis, inhibited differentiation, and reduced pERK and pCREB signaling and the expression of transcription factors Pax6 and Lhx2. GDE caused persistent spatial and social investigation memory deficits compared with saline controls, regardless of sex. Interestingly, GDE adult mice exhibited enhanced repetitive and anxiety-like behavior, irrespective of sex. GDE reduced synaptic plasticity-related protein expression and caused hippocampal synaptic plasticity (LTP and LTD) deficits in adult offspring. These findings demonstrate that binge-like ethanol exposure at the GD8 and GD12 developmental stages causes defects in pERK–pCREB signaling and reduces the expression of Pax6 and Lhx2, leading to impaired cellular differentiation during the embryonic stage. In the adult stage, binge-like ethanol exposure caused persistent synaptic and behavioral abnormalities in adult mice. Furthermore, the findings suggest that combining ethanol exposure at two sensitive stages (GD8 and GD12) causes deficits in synaptic plasticity-associated proteins (Arc, Egr1, Fgf1, GluR1, and GluN1), leading to persistent FASD-like neurobehavioral deficits in mice.

## 1. Introduction

During pregnancy, maternal alcohol consumption causes a range of developmental abnormalities in offspring, broadly described as fetal alcohol spectrum disorders (FASDs). It was estimated that FASDs occur in 2% to 5% of live births in the United States [1] and that FASDs continue to be a leading health issue in Western countries. In 2016, the Centers for Disease Control (CDC) suggested that more than 3 million childbearing women may be in danger of exposing their developing embryos to the potentially damaging effects of alcohol [2]. Due to the pervasive nature of FASDs, the World Health Organization considers prenatal alcohol exposure to be an important preventable cause of intellectual disability in the Western world. Despite public health cautions, approximately one in eight pregnant women (approximately 500,000 per year) consume alcohol at certain stages of or throughout pregnancy, and approximately 80,000 of these women binge drink [3]. Children with FASDs exhibit physical, cognitive, and behavioral deficits, including impulsivity, response inhibition, attention, learning and memory deficits [4,5,6], intellectual disabilities, reduced IQ, and anxiety/depression [7,8,9,10]. Among the numerous potential harmful effects of ethanol exposure during fetal growth, damage to the developing brain and enduring neurobehavioral abnormalities are the most common [1,5]. Behavioral problems associated with impulsivity, response inhibition, attention, activity, learning, and memory are common among children with FASDs [6,11,12,13,14,15,16,17,18,19,20,21]. The severity of deficits due to prenatal ethanol exposure in children with FASDs is associated with the gestational stage and/or the timing and degree of alcohol exposure [22].

Many women continue with the regular consumption of alcohol before recognizing that they are pregnant [23,24]. However, by that time, a developing embryo will have already passed through two critical developmental stages: gastrulation in the third week of gestation and neurulation in the fourth week of gestation. Women of childbearing age drink higher amounts of alcohol before pregnancy recognition [25,26,27], with higher levels of binge drinking. Therefore, it is important to examine the impact of stage-specific gestational ethanol exposure on neurobehavioral impairments that are expressed in adulthood.

Several FASD animal models, established with varying ethanol doses at various developmental stages, have effectively recapitulated many of these behavioral deficits [28,29,30,31,32]. It was shown that embryos at certain gestational stages, such as gastrulation (GD7) and neurulation (GD8), are highly vulnerable to high-dose binge-like ethanol exposure [33,34]. Binge-like ethanol exposure on GD7 or GD8 caused damage to the fetal forebrain (frontal cortex, septal area, striatum, and hippocampus (HP)), as determined by histological and neuroimaging analyses [35,36,37,38,39,40,41,42,43]. Several rodent studies using similar acute high-dose binge-like ethanol (2.4 or 2.9 g/kg, twice at 4-h intervals) exposure during gastrulation and neurulation reported deficits in various spatial learning and memory tasks [44,45,46,47] and active and passive avoidance tasks [48]. However, a similar model in mice (2.8 g/kg twice at a 4-h interval) showed no ethanol effect in an object placement spatial memory task [49]. These high-dose models have been associated with higher levels of prenatal and perinatal death when compared with lower doses.

In another model, animals were exposed to ethanol vapor for 6 h, resulting in a blood ethanol concentration (BEC) of 88 mg/dL, which caused significantly increased anxiety-like behavior only in male adolescent offspring; the opposite effect was observed in males in adulthood, and female offspring were unaffected in both adolescence and adulthood [50]. There were also social deficits in male offspring, as well as substantial decreases in social preference in late adolescence and adulthood, regardless of sex [51]. These findings emphasized the possibility of risk associated with high-to-low binge-like alcohol exposure during the first month of human pregnancy. Despite the limitations of studies of human prenatal alcohol exposure (unknowns related to the pregnancy stage of alcohol exposure, amount of alcohol consumed, etc.) and undefined mechanisms, these studies highlighted the possible relationships between gestational-induced deficits in mice and humans.

Gastrulation, which corresponds to the third week of gestation in humans and GD6.25 to GD9. 5 in mice, is a critical developmental event during which embryonic cells undergo a series of differentiation toward an adult organism. Therefore, alcohol exposure during the early stages of embryonic development may produce many features of FASDs [34,52]. Furthermore, studies [34,52] have shown that early pregnancy is a sensitive period for alcohol-induced developmental disruptions. However, the effects on the fetus of combined alcohol exposure at these two sensitive stages (GD8 and GD12) are unknown. None of the gestational ethanol exposure studies have examined the association between molecular and behavioral outcomes in a single analysis. Therefore, in the current study, we modeled early gestational binge-like ethanol consumption by exposing GD8 and GD12 dams to ethanol and examining its impact on the forebrains of embryo (GD13.5) and adult mice.

Although the mechanism by which prenatal ethanol causes persistent behavioral deficits is unknown, several recent ethanol studies, based on different developmental stages, began to show the possible involvement of many synaptic plasticity-related proteins that have a well-established role in long-term memory formation [53,54,55]. Increased intracellular Ca^2+^ initiates a series of events that activate transcription factors, such as the cAMP response element-binding protein (CREB), in the nucleus. CREB binds to multiple recognition sites, including the cAMP response element (CRE), causing the induction of plasticity-related genes [56,57,58]. Together with other researchers, we recently demonstrated that postnatal ethanol exposure disrupts CREB phosphorylation [32,54,55,59,60], which may result in the disruption of the expression of immediate early genes, such as the activity-regulated cytoskeleton-associated protein (*Arc*) and early growth-response 1 (Egr1) [32,54,60,61,62,63,64]. However, whether binge-like ethanol exposure on GD8 and GD12 (GDE) causes behavioral impairments, and whether this correlates with persistent synaptic protein deficits in adult mice, are unknown. Therefore, our current study expands the mouse gestational binge-ethanol paradigm and reports enhanced apoptosis, reduced differentiation, and disrupted phosphorylated extracellular signal-regulated kinase (pERK), pCREB, and transcription factors in embryo forebrains (GD13.5) that underlie the immediate adverse effects of GDE. We also provide evidence on the damaging impacts of GDE and show an association in adulthood between behavioral deficits and decreased expression of synaptic plasticity-related proteins in the HP.

## 2. Materials and Methods

### 2.1. Animals

C57BL/6J mice were bred at the animal facility of the Nathan Kline Institute (NKI) and maintained under standard laboratory conditions (a 12 h light/12 h dark cycle) with food and water available ad libitum. Our animal care and handling procedures followed the guidelines of the National Institutes of Health and were approved by the NKI’s Institutional Care and Use Committee (IACUC). A male mouse was placed with a female mouse from 8:00 a.m. to 9:00 a.m. Successful copulation, determined by the presence of a mating plug, was designated as gestational day zero (GD0). Individual pregnant females were weighed and placed in a single cage with regular nesting material.

### 2.2. Ethanol Administration

Saline or ethanol was administered to pregnant dams via intraperitoneal (i.p.) injections twice at a two-hour interval; the ethanol group (GDE) was given 1.75 g/kg (each dose) ethanol in saline, and the saline control group (GDS) was given only saline on GD8 and again at GD12, as previously described [49,65,66]. The BECs in the pregnant dam serum were measured using a standard alcohol dehydrogenase-based method [67]. All i.p. injections and blood collections were achieved with appropriate animal-handling procedures, and every precaution was taken to minimize the suffering of pregnant dams. In some studies, GD13.5 embryos were used. For adult studies, both GDS- and GDE-exposed male and female offspring were weaned after 21 days (four mice per cage), allowed to grow normally to adulthood, and randomly assigned for adult (P90) studies (5–10 mice/3–5 litters/group). Separate cohorts of animals are used for each biochemical, LTP, LTD, or behavioral analysis.

### 2.3. Protein Extraction, Electrophoresis, and Immunoblotting

For the immunoblot procedures, GD13.5 forebrains without olfactory bulbs or adult HP (dorsal and ventral from both the hemisphere) homogenates were prepared, as previously described [32,55]. In all immunoblot tests, the blots were stained with Ponceau S to confirm equal loading in each lane before further processing. The blots were incubated for 3 h at room temperature or overnight at 4 °C with the following individual primary antibodies: anti-rabbit cleaved caspase-3 (CC3) (Asp175) (polyclonal, #9661, 1:1000); anti-mouse-β-actin (monoclonal, #3700, 1:1000); anti-rabbit CREB (monoclonal, #9197, 1:1000); anti-rabbit phospho-CREB (monoclonal, #9198, 1:1000); anti-rabbit p44/42 MAPK (ERK1/2) (polyclonal, # 9102, 1:2000); anti-rabbit-phospho-p44/42 MAPK (polyclonal, # 9101, 1:1000); anti-rabbit GluA1 (monoclonal, #13185) from Cell Signaling (Danvers, MA, USA); anti-mouse-Arc (C-7, #sc-17839, 1:1000); anti-mouse-Egr1 (monoclonal, S-25, #sc-101033, 1:500) from Santa Cruz Biotechnology, Inc. (Santa Cruz, CA, USA); anti-rabbit nucleoprotein expressed in proliferative cells (Ki67) (polyclonal, #PA5-19462); anti-mouse LIM homeobox 2 (Lhx2) (monoclonal, # MA5-15834) from Thermo Fisher (Waltham, MA, USA); anti-rabbit Paired Box 6 (Pax6) (polyclonal, #12323-1-AP) from Proteintech (Rosemont, IL, USA); anti-rabbit fibroblast growth factor 1 (Fgf1) (polyclonal, #ab207321) from Abcam (Waltham, MA, USA); and anti-rabbit GluN1 (polyclonal, #05-432) from Millipore Sigma (Burlington, MA, USA). The blots were processed and analyzed using a procedure similar to one previously described [32,55]. Blots incubated only with the secondary antibody (goat anti-mouse peroxidase conjugate, #AP124P, 1:5000; goat anti-rabbit, #AP132P, 1:5000) (Millipore Sigma, Burlington, MA, USA) exhibited no bands. The data were standardized to total protein and β-actin to quantify proteins of interest.

### 2.4. Y-Maze Spatial Memory (SM) Task

Separate cohorts of adult male (*n* = 8 mice) and female (*n* = 8 mice) GDS and GDE mice (*n* = 8 mice/group) were subjected to the SM task, which was performed using a symmetrical Y-maze exactly as we previously described [32,55]. Mouse entry into one arm (the novel arm) of the Y-maze was hindered with a sheet of opaque paper throughout the duration of training (10 min). After a 24-h intertrial interval, the mice could explore all three arms (3 min; preference trial). The number of arm entries and the time spent in each arm were manually recorded from video footage by an observer who was blinded to the treatments of the mice. The discrimination ratios for arm entries and dwell time were calculated using the following formula for the preference for the novel arm over the familiar other arm: novel/(novel + other).

### 2.5. Social Recognition Memory (SRM)

Separate cohorts of adult male (*n* = 8 mice) and female (*n* = 8 mice) GDS and GDE mice (*n* = 8 mice/group) were subjected to the SRM task, which was performed as we previously described [68]. The social investigative behaviors that were scored were previously described and included the following: direct contact with the juvenile (3–4 weeks old) while inspecting any part of the body surface (including grooming, licking, and pawing); sniffing of the mouth, ears, tail, anogenital area; and close following (within 1 cm) of the juvenile [69,70]. The percentage of social investigation was calculated by dividing the investigation time during the second exposure by the initial investigation time × 100.

### 2.6. Elevated Plus Maze (EPM)

The EPM test was performed as previously described [71,72]. Briefly, the behavioral apparatus consisted of two open arms (5 cm width × 30 cm length) and two closed arms (5 cm width × 30 cm length) elevated 50 cm above the floor and dimly illuminated. Each mouse was individually presented in the center of the maze facing an open arm and allowed to freely explore for 5 min. The amount of time present with the head and four paws in the open arms and closed arms of the maze, as well as the number of entries into each arm, were measured over 5 min. The maze was cleaned with 70% ethanol after each test to prevent any influence of the previously tested mouse. The times present in the open and closed arms were manually measured by an experimenter who was blind to the treatment. The percentage of open and closed arm entries and the time spent in open arms were calculated (*n* = 8 mice/sex/group).

### 2.7. Marble Burying Test (MBT)

Behavior in the marble burying test (MBT) is a distinctive natural reflection of repetitive compulsive-like behavior [73,74]. The MBT was performed in a home cage where individual mice (*n* = 8 mice/sex/group) were placed with extra bedding and 25 marbles arranged in a 5 × 5 grid, as previously described [73,75]. Briefly, home cages were filled with fresh, regular mouse bedding material to a depth of 5 cm, and the bedding surface was leveled. Each mouse was individually placed for habituation. After 10 min, each individual mouse was removed, placed in its designated test cage with arranged marbles, and allowed to explore for 15 min. The number of marbles buried (>50% of the marble covered by the bedding) was recorded.

### 2.8. Nestlet Shredding Test (NST)

Behavior in the nestlet shredding test (NST) is an accurate reflection of repetitive compulsive-like behavior that spontaneously occurs in mice [73,74]. The NST was performed, as previously described [73,75], by placing one mouse (*n* = 8 mice/sex/group) into a regular cage containing mouse bedding material. Cotton fiber (nestlets) (5 cm × 5 cm, 5 mm thick, ~2.5 g each) was weighed using an analytical balance. The nestlet was placed on top of the bedding in each test cage, and the cage was closed with a filter-top cover. During the testing period, food and water were offered, and the mouse was left undisturbed for 30 minutes. After test completion, the mouse was removed and returned to its home cage. The remaining intact nestlet material was collected using forceps, allowed to dry overnight, and weighed. The percentage of nestlets shredded was calculated by determining the weight difference divided by the starting weight.

### 2.9. LTP and Long-Term Depression (LTD)

HP slices (400 μm) and electrophysiological recordings were performed using a typical and previously described procedure [32,55]. Briefly, immediately after preparation, the HP slices (*n* = 5 mice/group; 10 slices/group) were transferred to a recording chamber (29 °C) and perfused with artificial cerebrospinal fluid (ACSF; in mM: 124.0 NaCl, 4.4 KCl, 1.0 Na_2_HPO_4_, 25.0 NaHCO_3_, 2.0 MgSO_4_, 2.0 CaCl_2_, and 10.0 glucose, osmolarity 290–300) and continuously bubbled with 95% O_2_ and 5% CO_2_ for 90 min. Electrodes (stimulating and recording) were placed at the CA1 stratum radiatum, and CA1 field excitatory postsynaptic potentials (fEPSPs) were recorded. The stimulus voltages were plotted against the fEPSP slopes to measure basal synaptic transmission. A 10 min baseline was recorded at an intensity that evoked approximately 35% of the maximum evoked response. LTP was produced by tetanic stimulation of the Schaeffer collateral pathway (4 pulses at 100 Hz; bursts were repeated at 5 Hz, and each tetanus included three 10-burst trains separated by 15 s) [32]. Then, the fEPSP responses were recorded for 2 h. LTD was induced by low-frequency stimulation (LFS; 1 Hz, 900 pulses) [76]. Then, the fEPSP responses were recorded for 60 min. The fEPSP slope was expressed as a percentage of the baseline.

### 2.10. Statistical Analysis

Where possible, we sought to randomize and blind the sample data. The unblinded experimental data were analyzed in the same manner for all conditions to remove possible experimenter bias. All experiments were evaluated using equal numbers of mice per treatment. We used one-way analysis of variance (ANOVA) or two-way ANOVA with Bonferroni’s post hoc test to compare the data. In all the comparisons, *p* < 0.05 was considered to indicate statistical significance. Prism software (GraphPad, San Diego, CA, USA) was used for all statistical analyses. All the data were presented as the mean ± SEM.

## 3. Results

### 3.1. Effects of GDE Exposure on Body Weight and Litter Data

GD8 and GD12 ethanol exposure paradigms produced a maternal BEC of 300 ± 10 mg/dL one hour after the second administration, which decreased to 80 ± 5 mg/dL at 9 h. Because BEC is comparable to brain ethanol concentration, and maternal BEC is comparable to fetal BEC [77], brain ethanol concentration in GD8 or GD12 embryos was presumed to be similar to that of maternal BECs. Unlike GDS exposure, GDE exposure did not cause abnormalities related to the number of pups/litter, the sex ratio, or the average pup weight on P7. In addition, unlike GDS exposure, GDE exposure failed to affect body weight in male and female adults.

### 3.2. Effects of GDE Exposure on Embryonic Cell Proliferation and Survival

**Figure 1:**
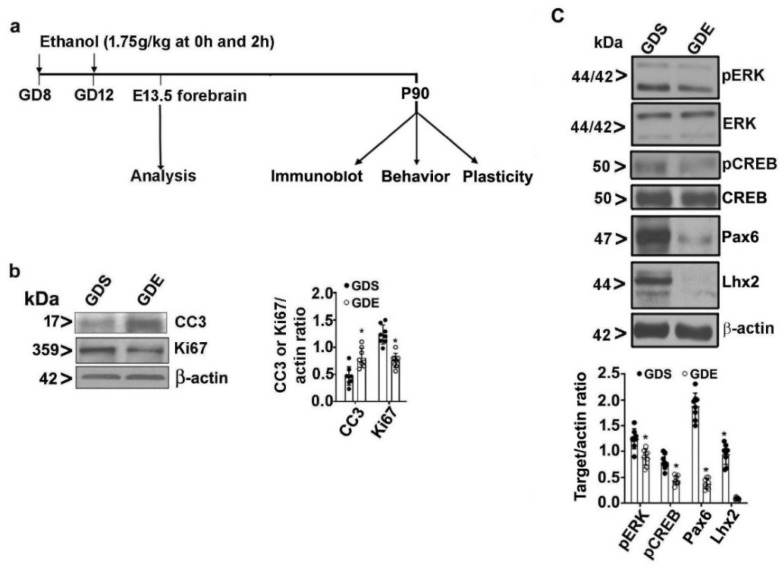
GDE enhances apoptosis and reduces proliferation in GD13.5 embryo forebrain tissues. Experimental design indicates developmental age, timing of ethanol exposure, and various analyses at early (GD13.5) and adult stages (**a**). Pregnant mice were exposed to 1.75 g/kg ethanol or saline i.p. at 0 h and again at 2 h on GD8 and GD12. Cleaved caspase-3 (CC3), a nucleoprotein expressed in proliferative cells (Ki67); (**b**) phosphorylated extracellular signal-regulated kinase (pERK), phosphorylated cAMP-response element binding protein (pCREB), Paired Box 6 (Pax6), and LIM homeobox 2 (Lhx2); (**c**) levels were evaluated in forebrain tissues obtained from GD13.5 embryos by Western blot analysis. The protein samples were equally loaded, confirmed with Ponceau S staining, and normalized to β-actin or total proteins. Error bars, SEM (* *p* < 0.05 vs. GDS group; *n* = 8 embryos/group) (one-way ANOVA).

The experimental design used in the present study is shown in Figure 1a. We examined neuronal stem cell (NSC) proliferation by subjecting GD13.5 embryo forebrain tissue extracts to immunoblotting with anti-Ki67 (a nucleoprotein expressed in proliferative cells) antibody. The Ki67 protein levels were significantly lower in the forebrain of GDE embryos than in the GDS controls on GD13.5 (F_3,21_ = 17; *p* < 0.05). We examined NSC apoptosis by evaluating the cleaved caspase-3 (CC3) levels using immunoblotting. The CC3 levels were significantly higher in the forebrains of GDE embryos than in the forebrains of GDS controls on GD13.5 (Figure 1b) (F_3,21_ = 23; *p* < 0.05). We examined ERK signaling by determining pERK and ERK levels using immunoblotting of forebrain tissue. The pERK levels were significantly lower in the forebrains of the GDE group than in the forebrains of the GDS control group (F_3,21_ = 13; *p* < 0.05). Furthermore, we determined CREB signaling by examining pCREB and CREB levels in forebrain tissue. The pCREB levels were significantly reduced in the forebrains of the GDE group, compared with the forebrains of the GDS control group (F_3,21_ = 15; *p* < 0.05). We also examined the effects of GDE on two important transcription factors, Pax6 and Lhx2. We found significantly lower Pax6 (F_3,21_ = 37; *p* < 0.05) and Lhx2 (F_3,21_ = 32; *p* < 0.05) in the forebrains of the GDE group than in the forebrains of GDS controls (Figure 1c). These data suggested that ethanol exposure on GD8 and GD12 resulted in abnormal cell proliferation and affected survival during neocortical development at GD13.5.

### 3.3. Effects of GDE Exposure on SM Performance

**Figure 2:**
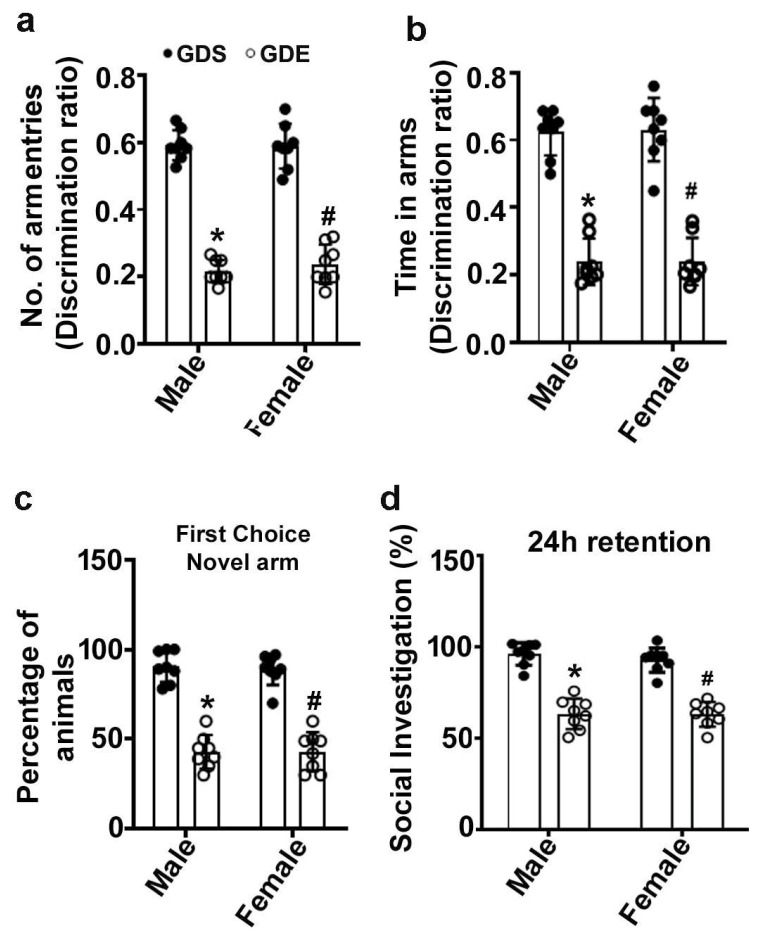
GDE-caused spatial memory and spatial recognition memory abnormalities in adult mice. SM was evaluated with a Y-maze in adult male and female mice exposed to GDS or GDE. The discrimination ratios (preference for the novel arm over the familiar other arm [novel/(novel + other)] for arm entries (**a**) and dwell time (time spent in each arm) (**b**) were determined for GDS and GDE mice 24 h after the first encounter with the partially opened maze. The percentages of mice choosing the novel arm as the first choice (**c**) are shown for GDS and GDE mice 24 h after the first encounter with the partially opened maze. The percentages of time spent in social investigation are shown (**d**) for the GDS and GDE groups 24 h after the first encounter by the same juvenile mice. Error bars, SEM (* *p* < 0.05 vs. GDS male group, # *p* < 0.05 vs. GDS female group; *n* = 8 mice/group/behavior).

Two-way ANOVA of data from the Y-maze test revealed that GDS-exposed male and female mice entered the novel, formerly unvisited arm of the maze more regularly and with longer dwell times than the previously visited arm (Figure 2a,b). However, GDE-exposed male and female mice displayed a significantly reduced preference for the novel arm (arm entry: male: F_3,21_ = 27; female: F_3,21_ = 20; *p* < 0.05) (Figure 2a) and spent less time in the novel arm (dwell time: male: F_3,21_ = 24, *p* < 0.05; female: F_3,21_ = 22; *p* < 0.05) (Figure 2b) than GDS-exposed mice after a 24 h retention period. Furthermore, the GDS-exposed male and female mice preferred the novel arm as the first choice, whereas the GDE-exposed male (F_3,21_ = 18, *p* < 0.05) and female (F_3,21_ = 16, *p* < 0.05) mice exhibited a significantly lower preference for the novel arm (Figure 2c). No significant (*p* > 0.05) difference was observed between male and female GDS or GDE mice.

### 3.4. Effects of GDE Treatment on SRM

Two-way ANOVA of the data from the SRM test revealed that GDE-treated male and female mice displayed significantly impaired SRM compared with the GDS-treated male (F_3,21_ = 16, *p* < 0.05) and female (F_3,21_ = 18, *p* < 0.05) animals (Figure 2d). Less than 3–4% of the animals exhibited aggressive encounters, and these mice were excluded from the statistical analysis. These results indicated that GDE exposure causes SRM impairments irrespective of sex (*p* > 0.05) in adult mice.

### 3.5. Effects of GDE Treatment on Stereotyped Repetitive Behavior in the NST and MBT

**Figure 3:**
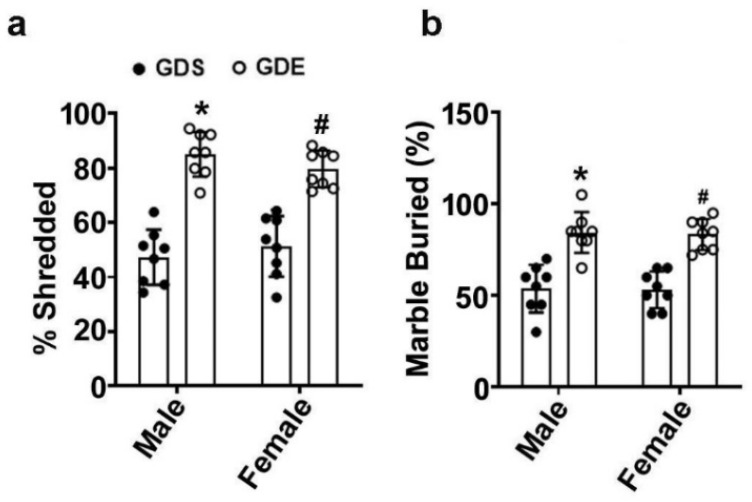
GDE-caused repetitive behavioral abnormalities in adult mice. The percentage of nestlets shredded was calculated by determining the weight difference divided by the starting weight in adult male and female mice exposed to GDS or GDE (**a**). The number of marbles buried (>50% marble enclosed by bedding) was recorded in adult male and female mice exposed to GDS or GDE (**b**). Error bars, SEM (* *p* < 0.05 vs. GDS male group, # *p* < 0.05 vs. GDS female group; *n* = 8 mice/group/behavior).

The effect of GDE exposure on repetitive behavior in the NST is shown in Figure 3a. Based on two-way ANOVA followed by post hoc analysis, GDE-exposed mice shredded significantly more nestlets than GDS-exposed male and female mice (male, F_3,21_ = 13; female, F_3,21_ = 12; *p* < 0.05). Notably, no significant difference in the percentage of shredded nestlets was observed between the male and female mice (*p* > 0.05). The effect of GDE treatment on the repetitive behavior in the MBT is shown in Figure 3b. Two-way ANOVA and post hoc analysis revealed that GDE-exposed mice exhibited a significantly higher percentage of buried marbles than GDS-exposed mice (male, F_3,21_ = 8; female, F_3,21_ = 9; *p* < 0.05). Notably, no significant difference was observed in the percentage of marbles buried by male and female mice (*p* > 0.05).

### 3.6. Effects of GDE Exposure on Anxiety-like Behavior in EPM

**Figure 4:**
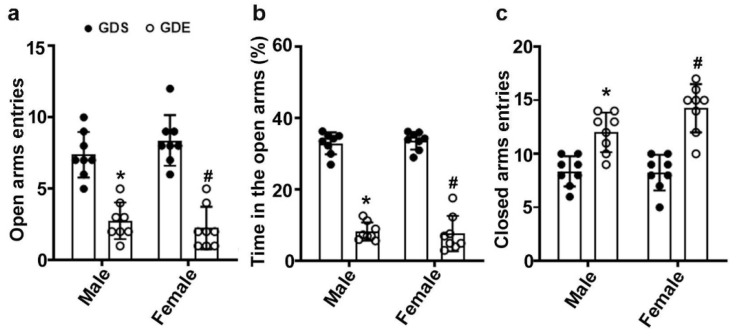
GDE-enhanced anxiety-like behaviors in adult male and female mice. Anxiety-like behavior in the elevated plus maze was calculated by determining the percentage of open arm entries (**a**), the percentage of open arm time (**b**), and closed arm entries (**c**) for adult males and females. Error bars, SEM (* *p* < 0.05 vs. GDS male group, # *p* < 0.05 vs. GDS female group; *n* = 8 mice/group).

Two-way ANOVA with the data from the EPM test revealed that GDE exposure, compared to GDS exposure, significantly reduced the percentage of open arm entries (male, F_3,21_ = 12; female, F_3,21_ = 11; *p* < 0.05) (Figure 4a) in tandem with an increase in closed arm entries (male, F_3,21_ = 6; female, F_3,21_ = 8; *p* < 0.05) (Figure 4b), and significantly less time in the open arms (male, F_3,21_ = 19; female, F_3,21_ = 16; *p* < 0.05) (Figure 4c). No significant effects of sex were found (*p* > 0.05).

### 3.7. Effects of GDE Treatment on LTP and LTD

**Figure 5:**
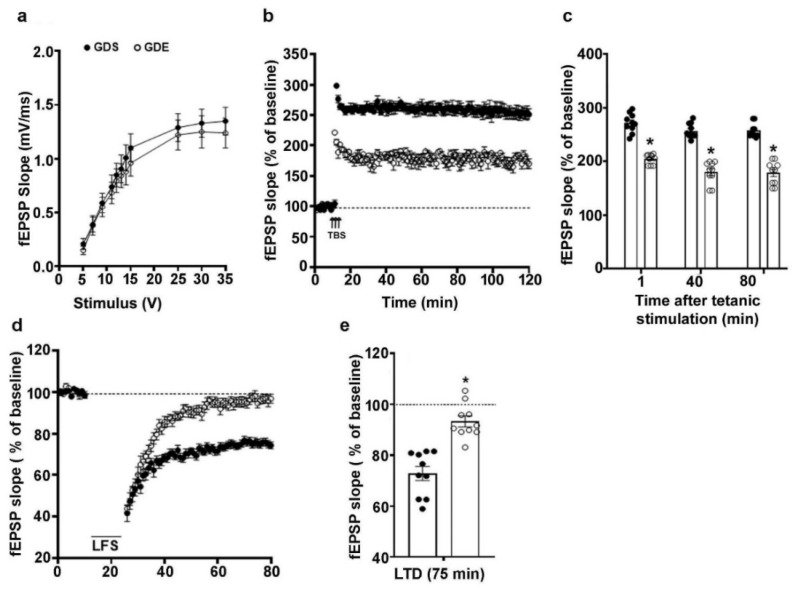
GDE-caused LTP and LTD impairments in adult mice. Input/output relationship plots of HP slices from GDS and GDE mice (**a**). The average field excitatory postsynaptic potentials (fEPSP) slope at various time points obtained from GDS and GDE adult male mice (**b**). For each slice, the fEPSP slopes were normalized against the average slope over the 10 min recording period before LTP induction. The arrows show the time of theta burst stimulation (TBS) (4 pulses at 100 Hz; the bursts repeated at 5 Hz, and each tetanus included three different 10-burst trains separated by 15 s). The bar graph indicates the average fEPSP slopes at multiple time points after TBS for the GDS and GDE groups (**c**). The average fEPSP slope at various time points obtained from GDS and GDE adult male mice after LTD induced by low-frequency stimulation (LFS) (**d**). The fEPSP slopes were normalized to the average value 10 min before LFS stimulation. A bar graph indicates a combined plot of the averages of fEPSP slopes at 75 min and shows the absence of LTD induced by LFS in GDE mice compared with GDS mice (**e**). Error bars, SEM (* *p* < 0.05 vs. GDS group; *n* = 5 mice/group; 10 slices/group).

The effects of GDE on LTP were assessed using male mice, as we found no sex effects in spatial memory or other behavioral tests. I/O responses were not significantly different between GDS- and GDE-exposed mouse HP slices (*p* > 0.05). The baseline fEPSP recording was performed for 10 min at 60 s intervals with a stimulation intensity equivalent to ~35% of the maximum evoked response. The application of theta burst stimulation (TBS) in HP slices from GDS-exposed mice produced strong LTP that was constant over 120 min. However, TBS application in HP slices from GDE mice resulted in a significantly reduced LTP magnitude (F_1,18_ = 33, *p* < 0.05; one-way ANOVA) (Figure 5a–c). The application of low-frequency stimulation to HP slices from GDS-exposed mice resulted in typical LTD. However, LFS in GDE HP slices resulted in defective LTD magnitudes (Figure 5d,e).

### 3.8. Effects of GDE on Synaptic Plasticity-Related Protein Expression in Adult Mice

**Figure 6:**
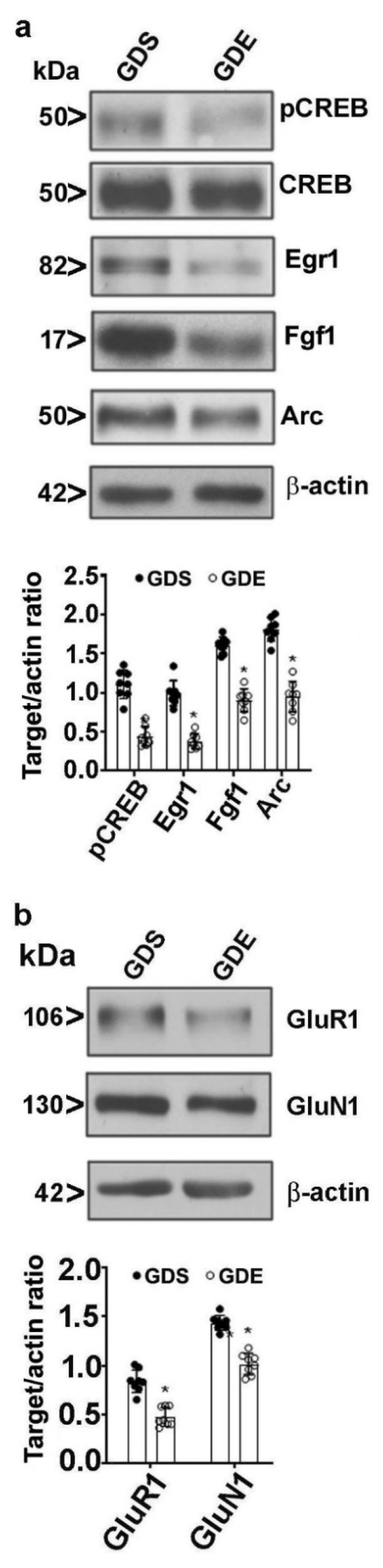
Decreased pCREB, Egr1, Fgf1, Arc (**a**), GluR1, and GluN1 (**b**) protein levels in adult mouse HP tissues in response to GDE. HP nuclear or membrane fractions were subjected to Western blot analysis. The protein samples were equally loaded, confirmed with Ponceau S staining, and normalized to β-actin and total proteins. Error bars, SEM (* *p* < 0.05 vs. GDS group; *n* = 8 mice/group).

We examined the persistent effects of GDE on synaptic plasticity-related proteins in the HP using immunoblotting. One-way ANOVA of the immunoblot data revealed significantly lower pCREB(F_3,21_ = 13; *p* < 0.05), Egr1(F_3,21_ = 26; *p* < 0.05), Fgf1(F_3,21_ = 37; *p* < 0.05), and Arc (F_3,21_ = 16; *p* < 0.05) protein levels in the adult GDE male HP than in the corresponding levels in the GDS control HP (Figure 6a). We also examined the persistent effects of GDE on α-amino-3-hydroxy-5-methyl-4-isoxazolepropionic acid (AMPA) (GluR1 subunit) and N-methyl-D-aspartate (NMDA) (GluN1 subunit) protein expression using immunoblotting. One-way ANOVA of the immunoblot data revealed significantly lower GluR1(F_3,21_ = 12; *p* < 0.05) and GluN1(F_3,21_ = 11; *p* < 0.05) protein levels in the adult GDE male HP than in the corresponding levels in the GDS control HP (Figure 6b).

## 4. Discussion

Human and preclinical animal studies have reported significant neurobehavioral abnormalities in offspring after prolonged low-to high-dose prenatal ethanol exposure, i.e., exposure resulting in low-to-high BECs [78,79,80,81,82,83]. However, limited animal studies have investigated the effects of lower, moderate, and high ethanol doses administered acutely during specific developmental stages [47,84,85,86] and established the time, pattern, and dose of ethanol exposure to model heavy human drinking (i.e., 3–5 drinks in one sitting on 2 successive days). This issue is especially important when women continue with a regular consumption of alcohol before recognizing that they are pregnant [23,24]. Drinking during this early unrecognized pregnancy period could expose a developing embryo at critical developmental stages, such as the gastrulation or neurulation periods of gestation [47,84,85,86]. Moreover, some women of childbearing age drink higher levels of alcohol before pregnancy recognition [25,26,27] and exhibit a higher proportion of binge drinking.

In the current study, we evaluated acute binge-like ethanol exposure at the early gestational period and modeled human drinking during the third and fifth weeks of pregnancy [87]. The current ethanol exposure paradigm resulted in maternal BECs of 300 mg/dL 1 h after the second dose, which reduced to 80 mg/dL at 9 h. This result caused no apparent deficits related to the number of pups/litters, sex ratio, or average pup weight on P7 compared to GDS-exposed females. The GDE- and GDS-exposed male and female adult animals exhibited normal body weight. Similar observations were found in previous studies based on acute early gestational ethanol exposure [50,51,66,88]. In contrast, chronic ethanol exposure throughout the gestational period has been shown to reduce pup or offspring body weight [89,90]. In the prenatal stage (GD13.5), GDE exposure induced cell death and reduced cell differentiation and cell survival signaling (pERK and pCREB). In addition, the expression of transcription factors Pax6 and Lhx2 was decreased in the GD13.5 forebrains of mice exposed to GDE. These results suggested that reduced-cell survival and cell-death events are immediate teratogenic results of early gestational ethanol exposure.

Previous studies have found excessive cell death due to ethanol exposure during the GD7 and GD14 stages [91,92,93]. However, the basis for the selective vulnerability to ethanol-induced cell death remains unclear. It is possible that GDE-induced inhibition of survival signaling (pERK and pCREB) could contribute to cell death. Indeed, previous studies have shown that ethanol exposure during neurogenesis perturbs the MAPK pathway and downstream effectors (pCREB) [94,95,96] and adversely affects cyclin D1, which aids the balance between neuronal differentiation and proliferation [97,98]. Similarly, reduced pERK and pCREB were also found in postnatal ethanol-exposed P7 mice, causing neuronal apoptosis [32,55,60]. Furthermore, GDE exposure reduced Pax6 expression in the forebrains of mice at GD13.5. Pax6 is a transcription factor that has an essential function in cell proliferation and is involved in the regional specification of the telencephalon. Consistent with our data, previous studies reported a reduced expression of Pax6 in response to ethanol exposure [99,100,101,102]; forced expression of Pax6 rescued ethanol-induced deficits in neuronal differentiation [101,103]. However, GDE exposure from GD6 to GD16 caused enhanced Pax6 expression in the E12 embryonic brains of mice [104].

We also found that GDE exposure reduced Lhx2 expression in the forebrains of mice at GD13.5. Lhx2, a transcription factor, plays an essential function in cortical development by determining cortical progenitor fate during the early stages of corticogenesis [105,106]. Two previous studies reported a lack of ethanol effect on Lhx2 transcripts in embryonic human-derived cultured cortical slices or mouse embryonic cortical tissues obtained from two different prenatal ethanol models (GD14 to GD16 [107] and GD0.5 to GD19.5 [108]. These inconsistent data may be due to the developmental stage at which ethanol exposure occurred, the cells that were impacted, and the tissue used for analysis. Moreover, we found no studies that analyzed Lhx2 protein expression in any prenatal ethanol studies. Taken together, our findings indicated that GDE exposure impairs transcription factors, such as Pax6 and Lhx2 expression, which have a critical function in neuronal differentiation.

It is well-established that the HP is one of the most vulnerable and affected structures following developmental ethanol exposure. Therefore, most studies have evaluated spatial memory to demonstrate disruption of the HP [32,64,109,110,111]. Previous studies using high ethanol doses administered over exposure times lasting a few or several days to a full trimester-equivalent or more showed impairments in spatial learning and memory on an array of tasks (e.g., radial arm maze, water maze, Y-maze, and T-maze alternation) in rodent offspring at different postnatal developmental stages (e.g., adolescent and adult) (for a review, see [28,112,113,114]). Collectively, these studies indicated that the extent of spatial memory deficits is related to dose, time, and mode of ethanol exposure and correlated to the age at which animals are examined [115]. Previous studies in which a high amount of ethanol exposure was restricted to a narrow window early in development, such as neurulation [116], have found that the exposure caused either no impairments [49], typical acquisition but impaired retention in spatial learning tasks [44], deficits in the acquisition of spatial learning tasks [46], or deficits in spatial reference memory [47]. It is unknown whether other studies [50,51] in which exposure was restricted to the early neurogenesis period (GD11 or GD12) [116] indicate that the exposure causes spatial memory deficits, although these studies have reported other behavioral deficits.

The current study reported a significant deficit in spatial memory in the Y-maze, where GDE significantly diminished the number of arm entries and time spent in the novel arm. In addition, a high percentage of GDE animals exhibited reduced entry into the novel arm as the first choice, compared with their GDS counterparts. These observations further emphasize the increased vulnerability of embryos in the neurulation and neurogenesis periods to ethanol-induced spatial memory impairments and suggested the importance of the duration of exposure in addition to the ethanol levels and the gestational timing of exposure. Consistent with these animal models, binge-like alcohol exposure during early pregnancy in primates produced significant behavioral impairments in offspring (for references, see [117]). In addition, a recent cross-sectional study reported that children with low levels of prenatal alcohol exposure exhibited significant structural abnormalities and more behavioral impairments than a well-matched control group [118]. These observations indicate that gestation alcohol exposure, even in small amounts, produces a measurable influence on brain structure and behavior in offspring.

Maternal ethanol has been shown to produce alterations in social behavior in exposed children. Children have difficulties contemplating the consequences of their actions, appreciating social cues, and interacting in social contexts [119,120,121]. Studies using animal models of developmental ethanol at different developmental stages, such as GDE [50,51,122,123,124,125,126] and postnatal [32,64,127,128] periods, or ethanol exposure across all three trimesters [129], have reported impaired social interaction in both males and females or impairment in a sexually dimorphic way [50,51,126,128]. In the current study, GDE exposure caused significantly reduced social interaction in male and female adult mice. In general, previous studies showed that social interaction deficits found in ethanol-exposed animals were observed at all ages [130]. However, in some studies, reduced social preference lessened with age and was no longer observed in P77 males [51].

Although cognitive deficits are the major outcome of prenatal ethanol exposure, increased anxiety behavior has also been observed in many FASDs [131,132,133,134] and animal studies [78,135,136,137,138]. However, some studies reported increased or decreased anxiety-like behaviors, depending on sex, following prenatal ethanol exposure [50,51,126,139,140,141,142]. In our study, GDE exposure significantly decreased open-arm entries in tandem with increased closed-arm entries. In addition, GDE animals spent significantly less time than GDS-exposed male and female adult mice in the open arms. We also found enhanced marble-burying behavior in GDE male and female mice. Previous studies have shown enhanced marble-burying behavior only in prenatal ethanol-exposed male offspring [50,143]. Administration of BDNF in offspring exposed to prenatal ethanol rescued marble-burying behavior [143]. Our data indicated enhanced nestlet shredding behavior in GDE-exposed male and female mice, consistent with the marble-burying behavior. Although these studies emphasized the importance of anxiety-like behavior and obsessive-compulsive disorder (OCD) in prenatal ethanol exposure, the reason for the inconsistent literature regarding the effects of prenatal ethanol exposure on anxiety-like behaviors in males and females is unknown. It is possible that the expression of anxiety-like behavior is very complex and may be influenced by many environmental factors, including the amounts of ethanol and the timing of ethanol exposure, secondary effects, and the developmental stages at which the behaviors were observed [115,144,145]. Taken together, these data suggested that binge-like ethanol exposure during GD 8 and GD12 predisposes animals to anxiety-like behavior and OCD.

Synaptic plasticity is the biological process through which synaptic activity results in synaptic strength and ultimately contributes to behavioral learning and memory processes. LTP is a type of synaptic plasticity in which synaptic connections become more robust after high-frequency stimulation (HFS). Our study and previous studies [32,64,146,147,148] of postnatal ethanol models indicated reduced LTP in the CA3-CA1 Schaffer collateral pathway in adult offspring. Prenatal ethanol exposure also caused reduced LTP in the HP [82,146,149,150]. Persistent changes in synaptic plasticity can also include a weakening of synaptic efficacy, which is referred to as LTD. However, very few studies have determined the effects of early prenatal ethanol on LTD in offspring. In a previous study, the exposure of dams to ethanol throughout the gestation period resulted in a reduced LTD only in male juvenile rat offspring [151]. However, ethanol exposure spanning all three trimesters caused enhanced LTD (LFS 600 × 1 Hz and paired pulses- LFS-200-900) in young adult rat hippocampal slices [152]. In our study, GDE exposure caused a reduced magnitude of LTD in both male and female adult offspring.

Thus, the current study indicates that GDE impairs bidirectional synaptic plasticity (LTP and LTD) in the CA3-CA1 Schaffer collateral pathway in both male and female adult offspring. HFS-induced LTP or LFS (900 pulses applied at 1 Hz)-induced LTD in the Schaffer collateral pathway has been previously shown to be dependent on N-methyl-D-aspartate receptors (NMDARs). Our current findings indicated a reduction in GluN1 in the HP. However, the effects of prenatal or postnatal ethanol exposure on NMDA receptor subunits have been inconclusive [153,154,155,156]. The findings cannot be compared, because each study used different ethanol exposure paradigms and different animal species. Therefore, although reduced LTP and LTD in the HP have been associated with reduced GluN1 levels after GDE, the possible link between these results and the expression levels of NMDA receptor subunits warrants further study in the future.

We also found that GDE exposure caused reduced GluR1 in the HP of adult offspring. A similar reduction in GluR1 expression was shown in the HP of female adult offspring exposed to ethanol throughout the gestation period [157,158]. Consistent with these changes, ethanol exposure throughout gestation impaired CA1 α-amino-3-hydroxy-5-methyl-4-isoxazolepropionic acid receptor (AMPAR)-mediated neurotransmission [159,160]. Because AMPAR regulation at the synaptic surface controls NMDAR-dependent synaptic plasticity at the CA3-CA1 Schaffer collateral pathway [161,162], reduced expression of calcium-permeable GluR1 receptors can produce changes in downstream signaling molecules, including pCREB [158,163], leading to impaired synaptic plasticity gene expression and synaptic plasticity [164]. These observations indicate that GDE may cause structural defects. Indeed, the exposure of pregnant rhesus macaques to ethanol (1.5 g/kg/d) over the first 60 days of gestation resulted in significantly induced structural abnormalities in fetuses at gestational day 135, as measured by magnetic resonance image (MRI) [165]. These abnormalities were associated with the reduced excitatory, but not inhibitory, postsynaptic event amplitude in the somatosensory cortex and putamen [165], suggesting a functional significance of the structural effects of fetal alcohol exposure.

As several studies demonstrated, pCREB promotes the transcription of synaptic plasticity genes, which are essential in hippocampal function and in the process of memory formation [53,54,55]. Together with other researchers, we recently demonstrated that postnatal ethanol exposure disrupts CREB phosphorylation [32,54,55,59,60], which affects many synaptic proteins, including the expression of immediate early genes, such as Arc and Egr1 [32,54,60,64]. In addition, we and other researchers have reported decreased expression of synaptic plasticity (*Bdnf*, *C-Fos*, *Egr1*, and *Arc*)-related genes [64] and Egr1 and Arc protein expression [32,54,60,61,62,63,64] in different ethanol models. Consistent with our findings, a recent study of the mouse HP showed that Fgf1 enhances the maintenance of synaptic plasticity and improves associative contextual fear memory. Interestingly, temporal regulation of Fgf1b gene expression was shown to be correlated with the strength of associative memory, so that a weak training protocol leads to a transient increase in Fgf1, whereas strong training leads to a sustained increase in Fgf1 [166]. Consistent with these earlier studies, the current findings suggested reduced pCREB and expression of synaptic plasticity-related proteins, such as Egr1, Fgf1, and Arc, in the adult HP. Thus, the present results convincingly show that ethanol exposure on GD8 and GD12 can impact developmental programs and contribute to persistent synaptic plasticity gene expression and neurobehavioral deficits.

## 5. Conclusions

In summary, ethanol exposure during the active cell differentiation stage of brain development causes cell apoptosis, reduces cellular proliferation, and reduces survival signaling and transcription factor levels in embryos. In addition, GDE-induced impairments that occur within cells of the early-developing brain may delay or reduce synaptic circuit maturation, leading to persistent deficits in synaptic plasticity, learning and memory deficits, anxiety, and repetitive and OCD-like behavior in adults, which are similar to the effects observed among children with FASDs [1,5,7,167,168,169]. The current findings demonstrate that ethanol exposure at two sensitive stages (GD8 and GD12) causes changes in synaptic plasticity-related proteins (Arc, Egr1, Fgf1, GluR1, and GluN1) and FASD-like neurobehavioral deficits in mice.

## Data Availability

Not applicable.

## References

[1] Riley E.P., Infante M.A., Warren K.R. (2011). Fetal alcohol spectrum disorders: An overview. Neuropsychol. Rev..

[2] CDC (2016). Alcohol and Pregnancy. www.cdc.gov/vitalsigns/fasd.

[3] Floyd R.L., Weber M.K., Denny C., O’Connor M.J. (2009). Prevention of fetal alcohol spectrum disorders. Dev. Disabil. Res. Rev..

[4] Basavarajappa B.S. (2015). Fetal Alcohol Spectrum Disorder: Potential Role of Endocannabinoids Signaling. Brain Sci..

[5] Coles C.D., Goldstein F.C., Lynch M.E., Chen X., Kable J.A., Johnson K.C., Hu X. (2011). Memory and brain volume in adults prenatally exposed to alcohol. Brain Cogn..

[6] Graham D.M., Crocker N., Deweese B.N., Roesch S.C., Coles C.D., Kable J.A., May P.A., Kalberg W.O., Sowell E.R., Jones K.L. (2013). Prenatal alcohol exposure, attention-deficit/hyperactivity disorder, and sluggish cognitive tempo. Alcohol. Clin. Exp. Res..

[7] Green C.R., Mihic A.M., Nikkel S.M., Stade B.C., Rasmussen C., Munoz D.P., Reynolds J.N. (2009). Executive function deficits in children with fetal alcohol spectrum disorders (FASD) measured using the Cambridge Neuropsychological Tests Automated Battery (CANTAB). J. Child. Psychol. Psychiatry.

[8] Jacobson S.W., Jacobson J.L., Stanton M.E., Meintjes E.M., Molteno C.D. (2011). Biobehavioral markers of adverse effect in fetal alcohol spectrum disorders. Neuropsychol. Rev..

[9] Jones K.L., Hoyme H.E., Robinson L.K., del Campo M., Manning M.A., Prewitt L.M., Chambers C.D. (2010). Fetal alcohol spectrum disorders: Extending the range of structural defects. Am. J. Med. Genet. A.

[10] Lebel C., Mattson S.N., Riley E.P., Jones K.L., Adnams C.M., May P.A., Bookheimer S.Y., O’Connor M.J., Narr K.L., Kan E.Z. (2012). Sowell, A longitudinal study of the long-term consequences of drinking during pregnancy: Heavy In Utero alcohol exposure disrupts the normal processes of brain development. J. Neurosci..

[11] Bhatara V., Loudenberg R., Ellis R. (2006). Association of attention deficit hyperactivity disorder and gestational alcohol exposure: An exploratory study. J. Atten. Disord..

[12] Burden M.J., Jacobson S.W., Sokol R.J., Jacobson J.L. (2005). Effects of prenatal alcohol exposure on attention and working memory at 7.5 years of age. Alcohol. Clin. Exp. Res..

[13] Coles C.D., Platzman K.A., Raskind-Hood C.L., Brown R.T., Falek A., Smith I.E. (1997). A comparison of children affected by prenatal alcohol exposure and attention deficit, hyperactivity disorder. Alcohol. Clin. Exp. Res..

[14] Kaemingk K.L., Halverson P.T. (2000). Spatial memory following prenatal alcohol exposure: More than a material specific memory deficit. Child. Neuropsychol..

[15] Kaemingk K.L., Mulvaney S., Halverson P.T. (2003). Learning following prenatal alcohol exposure: Performance on verbal and visual multitrial tasks. Arch. Clin. Neuropsychol..

[16] O’Malley K.D., Nanson J. (2002). Clinical implications of a link between fetal alcohol spectrum disorder and attention-deficit hyperactivity disorder. Can. J. Psychiatry.

[17] Streissguth A.P., O’Malley K. (2000). Neuropsychiatric implications and long-term consequences of fetal alcohol spectrum disorders. Semin. Clin. Neuropsychiatry.

[18] Streissguth A.P., Sampson P.D., Olson H.C., Bookstein F.L., Barr H.M., Scott M., Feldman J., Mirsky A.F. (1994). Maternal drinking during pregnancy: Attention and short-term memory in 14-year-old offspring—A longitudinal prospective study. Alcohol. Clin. Exp. Res..

[19] Uecker A., Nadel L. (1996). Spatial locations gone awry: Object and spatial memory deficits in children with fetal alcohol syndrome. Neuropsychologia.

[20] Uecker A., Nadel L. (1998). Spatial but not object memory impairments in children with fetal alcohol syndrome. Am. J. Ment. Retard..

[21] Willford J.A., Richardson G.A., Leech S.L., Day N.L. (2004). Verbal and visuospatial learning and memory function in children with moderate prenatal alcohol exposure. Alcohol. Clin. Exp. Res..

[22] O’Leary C.M., Nassar N., Kurinczuk J.J., de Klerk N., Geelhoed E., Elliott E.J., Bower C. (2010). Prenatal alcohol exposure and risk of birth defects. Pediatrics.

[23] Finer L.B., Zolna M.R. (2011). Unintended pregnancy in the United States: Incidence and disparities, 2006. Contraception.

[24] Quinn D.A., Sileanu F.E., Zhao X., Mor M.K., Judge-Golden C., Callegari L.S., Borrero S. (2020). History of unintended pregnancy and patterns of contraceptive use among racial and ethnic minority women veterans. Am. J. Obstet. Gynecol..

[25] Roberts S.C., Wilsnack S.C., Foster D.G., Delucchi K.L. (2014). Alcohol use before and during unwanted pregnancy. Alcohol. Clin. Exp. Res..

[26] Strandberg-Larsen K., Nielsen N.R., Andersen A.M.N., Olsen J., Gronbaek M. (2008). Characteristics of women who binge drink before and after they become aware of their pregnancy. Eur. J. Epidemiol..

[27] Tough S., Tofflemire K., Clarke M., Newburn-Cook C. (2006). Do women change their drinking behaviors while trying to conceive? An opportunity for preconception counseling. Clin. Med. Res..

[28] Berman R.F., Hannigan J.H. (2000). Effects of prenatal alcohol exposure on the hippocampus: Spatial behavior, electrophysiology, and neuroanatomy. Hippocampus.

[29] Brown K.L., Calizo L.H., Goodlett C.R., Stanton M.E. (2007). Neonatal alcohol exposure impairs acquisition of eyeblink conditioned responses during discrimination learning and reversal in weanling rats. Dev. Psychobiol..

[30] Ieraci A., Herrera D.G. (2007). Single alcohol exposure in early life damages hippocampal stem/progenitor cells and reduces adult neurogenesis. Neurobiol. Dis..

[31] Ikonomidou C., Bittigau P., Ishimaru M.J., Wozniak D.F., Koch C., Genz K., Price M.T., Stefovska V., Horster F., Tenkova T. (2000). Ethanol-induced apoptotic neurodegeneration and fetal alcohol syndrome. Science.

[32] Joshi V., Subbanna S., Shivakumar M., Basavarajappa B.S. (2019). CB1R regulates CDK5 signaling and epigenetically controls Rac1 expression contributing to neurobehavioral abnormalities in mice postnatally exposed to ethanol. Neuropsychopharmacology.

[33] Sulik K.K., Johnston M.C., Webb M.A. (1981). Fetal alcohol syndrome: Embryogenesis in a mouse model. Science.

[34] Webster W.S., Walsh D.A., Lipson A.H., McEwen S.E. (1980). Teratogenesis after acute alcohol exposure in inbred and outbred mice. Neurobehav. Toxicol..

[35] Ashwell K.W., Zhang L.L. (1996). Forebrain hypoplasia following acute prenatal ethanol exposure: Quantitative analysis of effects on specific forebrain nuclei. Pathology.

[36] Dunty W.C., Zucker R.M., Sulik K.K. (2002). Hindbrain and cranial nerve dysmorphogenesis result from acute maternal ethanol administration. Dev. Neurosci..

[37] Godin E.A., O’Leary-Moore S.K., Khan A.A., Parnell S.E., Ament J.J., Dehart D.B., Johnson B.W., Johnson G.A., Styner M.A., Sulik K.K. (2010). Magnetic resonance microscopy defines ethanol-induced brain abnormalities in prenatal mice: Effects of acute insult on gestational day 7. Alcohol. Clin. Exp. Res..

[38] Kotch L.E., Sulik K.K. (1992). Experimental fetal alcohol syndrome: Proposed pathogenic basis for a variety of associated facial and brain anomalies. Am. J. Med. Genet..

[39] Lipinski R.J., Hammond P., O’Leary-Moore S.K., Ament J.J., Pecevich S.J., Jiang Y., Budin F., Parnell S.E., Suttie M., Godin E.A. (2012). Ethanol-induced face-brain dysmorphology patterns are correlative and exposure-stage dependent. PLoS ONE.

[40] Schambra U.B., Lauder J.M., Petrusz P., Sulik K.K. (1990). Development of neurotransmitter systems in the mouse embryo following acute ethanol exposure: A histological and immunocytochemical study. Int. J. Dev. Neurosci..

[41] Sulik K.K. (1984). Craniofacial defects from genetic and teratogen-induced deficiencies in presomite embryos. Birth. Defects. Orig. Artic. Ser..

[42] Sulik K.K. (2005). Genesis of alcohol-induced craniofacial dysmorphism. Exp. Biol. Med..

[43] Sulik K.K., Johnston M.C., Daft P.A., Russell W.E., Dehart D.B. (1986). Fetal alcohol syndrome and DiGeorge anomaly: Critical ethanol exposure periods for craniofacial malformations as illustrated in an animal model. Am. J. Med. Genet. Suppl..

[44] Dumas R.M., Rabe A. (1994). Augmented memory loss in aging mice after one embryonic exposure to alcohol. Neurotoxicol. Teratol..

[45] Endres M., Toso L., Roberson R., Park J., Abebe D., Poggi S., Spong C.Y. (2005). Prevention of alcohol-induced developmental delays and learning abnormalities in a model of fetal alcohol syndrome. Am. J. Obstet. Gynecol..

[46] Summers B.L., Henry C.M., Rofe A.M., Coyle P. (2008). Dietary zinc supplementation during pregnancy prevents spatial and object recognition memory impairments caused by early prenatal ethanol exposure. Behav. Brain Res..

[47] Schambra U.B., Lewis C.N., Harrison T.A. (2017). Deficits in spatial learning and memory in adult mice following acute, low or moderate levels of prenatal ethanol exposure during gastrulation or neurulation. Neurotoxicol. Teratol..

[48] Molina J.C., Moyano H.F., Spear L.P., Spear N.E. (1984). Acute alcohol exposure during gestational day 8 in the rat: Effects upon physical and behavioral parameters. Alcohol.

[49] Sadrian B., Lopez-Guzman M., Wilson D.A., Saito M. (2014). Distinct neurobehavioral dysfunction based on the timing of developmental binge-like alcohol exposure. Neuroscience.

[50] Rouzer S.K., Cole J.M., Johnson J.M., Varlinskaya E.I., Diaz M.R. (2017). Moderate Maternal Alcohol Exposure on Gestational Day 12 Impacts Anxiety-Like Behavior in Offspring. Front. Behav. Neurosci..

[51] Diaz M.R., Mooney S.M., Varlinskaya E.I. (2016). Acute prenatal exposure to ethanol on gestational day 12 elicits opposing deficits in social behaviors and anxiety-like behaviors in Sprague Dawley rats. Behav. Brain Res..

[52] Webster W.S., Walsh D.A., McEwen S.E., Lipson A.H. (1983). Some teratogenic properties of ethanol and acetaldehyde in C57BL/6J mice: Implications for the study of the fetal alcohol syndrome. Teratology.

[53] Perissi V., Dasen J.S., Kurokawa R., Wang Z., Korzus E., Rose D.W., Glass C.K., Rosenfeld M.G. (1999). Factor-specific modulation of CREB-binding protein acetyltransferase activity. Proc. Natl. Acad Sci. USA.

[54] Subbanna S., Joshi V., Basavarajappa B.S. (2018). Activity-dependent Signaling and Epigenetic Abnormalities in Mice Exposed to Postnatal Ethanol. Neuroscience.

[55] Subbanna S., Nagre N.N., Umapathy N.S., Pace B.S., Basavarajappa B.S. (2015). Ethanol exposure induces neonatal neurodegeneration by enhancing CB1R Exon1 histone H4K8 acetylation and up-regulating CB1R function causing neurobehavioral abnormalities in adult mice. Int. J. Neuro Psychopharmacol..

[56] Bito H., Deisseroth K., Tsien R.W. (1996). CREB phosphorylation and dephosphorylation: A Ca (2+)- and stimulus duration-dependent switch for hippocampal gene expression. Cell.

[57] Lonze B.E., Ginty D.D. (2002). Function and regulation of CREB family transcription factors in the nervous system. Neuron.

[58] Wheeler D.G., Groth R.D., Ma H., Barrett C.F., Owen S.F., Safa P., Tsien R.W. (2012). Ca (V) 1 and Ca (V) 2 channels engage distinct modes of Ca (2+) signaling to control CREB-dependent gene expression. Cell.

[59] Krahe T.E., Wang W., Medina A.E. (2009). Phosphodiesterase inhibition increases CREB phosphorylation and restores orientation selectivity in a model of fetal alcohol spectrum disorders. PLoS ONE.

[60] Subbanna S., Nagre N.N., Shivakumar M., Joshi V., Psychoyos D., Kutlar A., Umapathy N.S., Basavarajappa B.S. (2018). CB1R-Mediated Activation of Caspase-3 Causes Epigenetic and Neurobehavioral Abnormalities in Postnatal Ethanol-Exposed Mice. Front. Mol. Neurosci..

[61] Heroux N.A., Horgan C.J., Rosen J.B., Stanton M.E. (2019). Cholinergic rescue of neurocognitive insult following third-trimester equivalent alcohol exposure in rats. Neurobiol. Learn. Mem..

[62] Heroux N.A., Robinson-Drummer P.A., Kawan M., Rosen J.B., Stanton M.E. (2019). Neonatal ethanol exposure impairs long-term context memory formation and prefrontal immediate early gene expression in adolescent rats. Behav. Brain Res..

[63] Jablonski S.A., Robinson-Drummer P.A., Schreiber W.B., Asok A., Rosen J.B., Stanton M.E. (2018). Impairment of the context preexposure facilitation effect in juvenile rats by neonatal alcohol exposure is associated with decreased Egr-1 mRNA expression in the prefrontal cortex. Behav. Neurosci..

[64] Shivakumar M., Subbanna S., Joshi V., Basavarajappa B.S. (2020). Postnatal Ethanol Exposure Activates HDAC-Mediated Histone Deacetylation, Impairs Synaptic Plasticity Gene Expression and Behavior in Mice. Int. J. Neuropsychopharmacol..

[65] Fish E.W., Wieczorek L.A., Rumple A., Suttie M., Moy S.S., Hammond P., Parnell S.E. (2018). The enduring impact of neurulation stage alcohol exposure: A combined behavioral and structural neuroimaging study in adult male and female C57BL/6J mice. Behav. Brain Res..

[66] White S.A., Weber J.N., Howard C.D., Favero C.B. (2015). Effects of binge ethanol exposure during first-trimester equivalent on corticothalamic neurons in Swiss Webster outbred mice. Neuroreport.

[67] Lundquist F. (1959). The determination of ethyl alcohol in blood and tissue. Meth. Biochem. Analy..

[68] Subbanna S., Basavarajappa B.S. (2014). Pre-administration of G9a/GLP inhibitor during Synaptogenesis Prevents Postnatal Ethanol-induced LTP Deficits and Neurobehavioral Abnormalities in Adult Mice. Exp. Neurol..

[69] Kogan J.H., Frankland P.W., Silva A.J. (2000). Long-term memory underlying hippocampus-dependent social recognition in mice. Hippocampus.

[70] Thor D.H., Wainwright K.L., Holloway W.R. (1982). Persistence of attention to a novel conspecific: Some developmental variables in laboratory rats. Dev. Psychobiol..

[71] Bahi A. (2013). Individual differences in elevated plus-maze exploration predicted higher ethanol consumption and preference in outbred mice. Pharmacol. Biochem. Behav..

[72] Bahi A., Dreyer J.L. (2014). Chronic psychosocial stress causes delayed extinction and exacerbates reinstatement of ethanol-induced conditioned place preference in mice. Psychopharmacology.

[73] Angoa-Perez M., Kane M.J., Briggs D.I., Francescutti D.M., Kuhn D.M. (2013). Marble burying and nestlet shredding as tests of repetitive, compulsive-like behaviors in mice. J. Vis. Exp..

[74] Thomas A., Burant A., Bui N., Graham D., Yuva-Paylor L.A., Paylor R. (2009). Marble burying reflects a repetitive and perseverative behavior more than novelty-induced anxiety. Psychopharmacology.

[75] Eissa N., Jayaprakash P., Azimullah S., Ojha S.K., Al-Houqani M., Jalal F.Y., Lazewska D., Kiec-Kononowicz K., Sadek B. (2018). The histamine H3R antagonist DL77 attenuates autistic behaviors in a prenatal valproic acid-induced mouse model of autism. Sci. Rep..

[76] Pensalfini A., Kim S., Subbanna S., Bleiwas C., Goulbourne C.N., Stavrides P.H., Jiang Y., Lee J.H., Darji S., Pawlik M. (2020). Endosomal Dysfunction Induced by Directly Overactivating Rab5 Recapitulates Prodromal and Neurodegenerative Features of Alzheimer’s Disease. Cell Rep..

[77] Burd L., Blair J., Dropps K. (2012). Prenatal alcohol exposure, blood alcohol concentrations and alcohol elimination rates for the mother, fetus and newborn. J. Perinatol..

[78] Cullen C.L., Burne T.H., Lavidis N.A., Moritz K.M. (2013). Low dose prenatal ethanol exposure induces anxiety-like behaviour and alters dendritic morphology in the basolateral amygdala of rat offspring. PLoS ONE.

[79] Komada M., Hara N., Kawachi S., Kawachi K., Kagawa N., Nagao T., Ikeda Y. (2017). Mechanisms underlying neuro-inflammation and neurodevelopmental toxicity in the mouse neocortex following prenatal exposure to ethanol. Sci. Rep..

[80] Lee J., Lunde-Young R., Naik V., Ramirez J., Orzabal M., Ramadoss J. (2020). Chronic Binge Alcohol Exposure during Pregnancy Alters mTOR System in Rat Fetal Hippocampus. Alcohol. Clin. Exp. Res..

[81] Tyler C.R., Allan A.M. (2014). Prenatal alcohol exposure alters expression of neurogenesis-related genes in an Ex Vivo cell culture model. Alcohol.

[82] Valenzuela C.F., Morton R.A., Diaz M.R., Topper L. (2012). Does moderate drinking harm the fetal brain? Insights from animal models. Trends. Neurosci..

[83] Barbier E., Houchi H., Warnault V., Pierrefiche O., Daoust M., Naassila M. (2009). Effects of prenatal and postnatal maternal ethanol on offspring response to alcohol and psychostimulants in long evans rats. Neuroscience.

[84] Parnell S.E., Holloway H.E., Baker L.K., Styner M.A., Sulik K.K. (2014). Dysmorphogenic effects of first trimester-equivalent ethanol exposure in mice: A magnetic resonance microscopy-based study. Alcohol. Clin. Exp. Res..

[85] Schambra U.B., Nunley K., Harrison T.A., Lewis C.N. (2016). Consequences of low or moderate prenatal ethanol exposures during gastrulation or neurulation for open field activity and emotionality in mice. Neurotoxicol. Teratol..

[86] Sulik K.K., Johnston M.C. (1983). Sequence of developmental alterations following acute ethanol exposure in mice: Craniofacial features of the fetal alcohol syndrome. Am. J. Anat..

[87] Otis E.M., Brent R. (1954). Equivalent ages in mouse and human embryos. Anat. Rec..

[88] Schambra U.B., Goldsmith J., Nunley K., Liu Y., Harirforoosh S., Schambra H.M. (2015). Low and moderate prenatal ethanol exposures of mice during gastrulation or neurulation delays neurobehavioral development. Neurotoxicol. Teratol..

[89] Aglawe M.M., Kale M.B., Rahangdale S.R., Kotagale N.R., Umekar M.J., Taksande B.G. (2021). Agmatine improves the behavioral and cognitive impairments associated with chronic gestational ethanol exposure in rats. Brain Res. Bull..

[90] Dandekar M.P., Bharne A.P., Borkar P.D., Subhedar N.K., Kokare D.M. (2019). Maternal ethanol exposure reshapes CART system in the rat brain: Correlation with development of anxiety, depression and memory deficits. Neuroscience.

[91] Dunty W.C., Chen S.Y., Zucker R.M., Dehart D.B., Sulik K.K. (2001). Selective vulnerability of embryonic cell populations to ethanol-induced apoptosis: Implications for alcohol-related birth defects and neurodevelopmental disorder. Alcohol. Clin. Exp. Res..

[92] Sulik K.K., Lauder J.M., Dehart D.B. (1984). Brain malformations in prenatal mice following acute maternal ethanol administration. J. Devl. Neurosci..

[93] Kotch L.E., Sulik K.K. (1992). Patterns of ethanol-induced cell death in the developing nervous system of mice; neural fold states through the time of anterior neural tube closure. Int. J. Dev. Neurosci..

[94] Louis L.K., Gopurappilly R., Surendran H., Dutta S., Pal R. (2017). Transcriptional profiling of human neural precursors post alcohol exposure reveals impaired neurogenesis via dysregulation of ERK signaling and miR-145. J. Neurochem..

[95] Dong W., Wu Z., Xu L., Fang Y., Xu Y. (2014). Maternal supplementation of nucleotides improves the behavioral development of prenatal ethanol-exposed mice. Cogn. Affect. Behav. Neurosci..

[96] Naseer M.I., Lee H.Y., Ullah N., Ullah I., Park M.S., Kim M.O. (2011). siRNA-mediated GABA (B) receptor at early fetal rat brain upon acute and chronic ethanol exposure: Down regulation of PKA and p-CREB expression. Synapse.

[97] Lange C., Huttner W.B., Calegari F. (2009). Cdk4/cyclinD1 overexpression in neural stem cells shortens G1, delays neurogenesis, and promotes the generation and expansion of basal progenitors. Cell Stem. Cell..

[98] Zhang R.L., Zhang Z.G., Roberts C., LeTourneau Y., Lu M., Zhang L., Wang Y., Chopp M. (2008). Lengthening the G (1) phase of neural progenitor cells is concurrent with an increase of symmetric neuron generating division after stroke. J. Cereb. Blood Flow Metab..

[99] Aronne M.P., Guadagnoli T., Fontanet P., Evrard S.G., Brusco A. (2011). Effects of prenatal ethanol exposure on rat brain radial glia and neuroblast migration. Exp. Neurol..

[100] Aronne M.P., Evrard S.G., Mirochnic S., Brusco A. (2008). Prenatal ethanol exposure reduces the expression of the transcriptional factor Pax6 in the developing rat brain. Ann. N. Y. Acad. Sci..

[101] Peng Y., Yang P.H., Ng S.S., Wong O.G., Liu J., He M.L., Kung H.F., Lin M.C. (2004). A critical role of Pax6 in alcohol-induced fetal microcephaly. Neurobiol. Dis..

[102] Wentzel P., Eriksson U.J. (2008). Genetic influence on dysmorphogenesis in embryos from different rat strains exposed to ethanol in vivo and In Vitro. Alcohol. Clin. Exp. Res..

[103] Mo Z., Milivojevic V., Zecevic N. (2012). Enforced Pax6 expression rescues alcohol-induced defects of neuronal differentiation in cultures of human cortical progenitor cells. Alcohol. Clin. Exp. Res..

[104] Kim K.C., Go H.S., Bak H.R., Choi C.S., Choi I., Kim P., Han S.H., Han S.M., Shin C.Y., Ko K.H. (2010). Prenatal exposure of ethanol induces increased glutamatergic neuronal differentiation of neural progenitor cells. J. Biomed. Sci..

[105] Bulchand S., Grove E.A., Porter F.D., Tole S. (2001). LIM-homeodomain gene Lhx2 regulates the formation of the cortical hem. Mech. Dev..

[106] Chou S.J., Perez-Garcia C.G., Kroll T.T., O’Leary D.D. (2009). Lhx2 specifies regional fate in Emx1 lineage of telencephalic progenitors generating cerebral cortex. Nat. Neurosci..

[107] Hashimoto-Torii K., Kawasawa Y.I., Kuhn A., Rakic P. (2011). Combined transcriptome analysis of fetal human and mouse cerebral cortex exposed to alcohol. Proc. Natl. Acad Sci. USA.

[108] Abbott C.W., Rohac D.J., Bottom R.T., Patadia S., Huffman K.J. (2018). Prenatal Ethanol Exposure and Neocortical Development: A Transgenerational Model of FASD. Cereb. Cortex..

[109] Cantacorps L., Alfonso-Loeches S., Moscoso-Castro M., Cuitavi J., Gracia-Rubio I., Lopez-Arnau R., Escubedo E., Guerri C., Valverde O. (2017). Maternal alcohol binge drinking induces persistent neuroinflammation associated with myelin damage and behavioural dysfunctions in offspring mice. Neuropharmacology.

[110] Gil-Mohapel J., Boehme F., Kainer L., Christie B.R. (2010). Hippocampal cell loss and neurogenesis after fetal alcohol exposure: Insights from different rodent models. Brain Res. Rev..

[111] Wagner J.L., Zhou F.C., Goodlett C.R. (2014). Effects of one- and three-day binge alcohol exposure in neonatal C57BL/6 mice on spatial learning and memory in adolescence and adulthood. Alcohol.

[112] Almeida L., Andreu-Fernandez V., Navarro-Tapia E., Aras-Lopez R., Serra-Delgado M., Martinez L., Garcia-Algar O., Gomez-Roig M.D. (2020). Murine Models for the Study of Fetal Alcohol Spectrum Disorders: An Overview. Front. Pediatr..

[113] Basavarajappa B.S., Subbanna S. (2016). Epigenetic Mechanisms in Developmental Alcohol-Induced Neurobehavioral Deficits. Brain Sci..

[114] Noor S., Milligan E.D. (2018). Lifelong Impacts of Moderate Prenatal Alcohol Exposure on Neuroimmune Function. Front Immunol..

[115] Marquardt K., Brigman J.L. (2016). The impact of prenatal alcohol exposure on social, cognitive and affective behavioral domains: Insights from rodent models. Alcohol.

[116] Finlay B.L., Darlington R.B. (1995). Linked regularities in the development and evolution of mammalian brains. Science.

[117] Schneider M.L., Moore C.F., Adkins M.M. (2011). The effects of prenatal alcohol exposure on behavior: Rodent and primate studies. Neuropsychol. Rev..

[118] Long X., Lebel C. (2022). Evaluation of Brain Alterations and Behavior in Children with Low Levels of Prenatal Alcohol Exposure. JAMA Netw. Open.

[119] Kelly S.J., Day N., Streissguth A.P. (2000). Effects of prenatal alcohol exposure on social behavior in humans and other species. Neurotoxicol. Teratol..

[120] Olson H.C., Feldman J.J., Streissguth A.P., Sampson P.D., Bookstein F.L. (1998). Neuropsychological deficits in adolescents with fetal alcohol syndrome: Clinical findings. Alcohol. Clin. Exp. Res..

[121] Thomas S.E., Kelly S.J., Mattson S.N., Riley E.P. (1998). Comparison of social abilities of children with fetal alcohol syndrome to those of children with similar IQ scores and normal controls. Alcohol. Clin. Exp. Res..

[122] Hamilton D.A., Akers K.G., Rice J.P., Johnson T.E., Candelaria-Cook F.T., Maes L.I., Rosenberg M., Valenzuela C.F., Savage D.D. (2010). Prenatal exposure to moderate levels of ethanol alters social behavior in adult rats: Relationship to structural plasticity and immediate early gene expression in frontal cortex. Behav. Brain Res..

[123] Hamilton D.A., Barto D., Rodriguez C.I., Magcalas C.M., Fink B.C., Rice J.P., Bird C.W., Davies S., Savage D.D. (2014). Effects of moderate prenatal ethanol exposure and age on social behavior, spatial response perseveration errors and motor behavior. Behav. Brain Res..

[124] Vega M.S.C., Chong S., Burne T.H. (2013). Early gestational exposure to moderate concentrations of ethanol alters adult behaviour in C57BL/6J mice. Behav. Brain Res..

[125] Shukla P.K., Meena A.S., Rao R., Rao R. (2018). Deletion of TLR-4 attenuates fetal alcohol exposure-induced gene expression and social interaction deficits. Alcohol.

[126] Varlinskaya E.I., Mooney S.M. (2014). Acute exposure to ethanol on gestational day 15 affects social motivation of female offspring. Behav. Brain Res..

[127] Boschen K.E., Hamilton G.F., Delorme J.E., Klintsova A.Y. (2014). Activity and social behavior in a complex environment in rats neonatally exposed to alcohol. Alcohol.

[128] Kelly S.J., Dillingham R.R. (1994). Sexually dimorphic effects of perinatal alcohol exposure on social interactions and amygdala DNA and DOPAC concentrations. Neurotoxicol. Teratol..

[129] Lugo J.N., Marino M.D., Cronise K., Kelly S.J. (2003). Effects of alcohol exposure during development on social behavior in rats. Physiol. Behav..

[130] Mooney S.M., Varlinskaya E.I. (2011). Acute prenatal exposure to ethanol and social behavior: Effects of age, sex, and timing of exposure. Behav. Brain Res..

[131] Hayes N., Moritz K.M., Reid N. (2020). Parent-reported sleep problems in school-aged children with fetal alcohol spectrum disorder: Association with child behaviour, caregiver, and family functioning. Sleep Med..

[132] O’Connor M.J., Paley B. (2009). Psychiatric conditions associated with prenatal alcohol exposure. Dev. Disabil. Res. Rev..

[133] Popova S., Temple V., Dozet D., O’Hanlon G., Toews C., Rehm J. (2021). Health, social and legal outcomes of individuals with diagnosed or at risk for fetal alcohol spectrum disorder: Canadian example. Drug Alcohol Depend..

[134] Schmidt N.B., Buckner J.D., Keough M.E. (2007). Anxiety sensitivity as a prospective predictor of alcohol use disorders. Behav. Modif..

[135] Brocardo P.S., Boehme F., Patten A., Cox A., Gil-Mohapel J., Christie B.R. (2012). Anxiety- and depression-like behaviors are accompanied by an increase in oxidative stress in a rat model of fetal alcohol spectrum disorders: Protective effects of voluntary physical exercise. Neuropharmacology.

[136] He F. (2014). The relationship of prenatal ethanol exposure and anxiety-related behaviors and central androgen receptor and vasopressin expression in adult male mandarin voles. Neuroscience.

[137] Liang J., Shen Y., Shao X.M., Scott M.B., Ly E., Wong S., Nguyen A., Tan K., Kwon B., Olsen R.W. (2014). Dihydromyricetin prevents fetal alcohol exposure-induced behavioral and physiological deficits: The roles of GABAA receptors in adolescence. Neurochem. Res..

[138] Wille-Bille A., Miranda-Morales R.S., Pucci M., Bellia F., D’Addario C., Pautassi R.M. (2018). Prenatal ethanol induces an anxiety phenotype and alters expression of dynorphin & nociceptin/orphanin FQ genes. Prog. Neuro Psychopharmacol. Biol. Psychiatry.

[139] Carneiro L.M., Diogenes J.P., Vasconcelos S.M., Aragao G.F., Noronha E.C., Gomes P.B., Viana G.S. (2005). Behavioral and neurochemical effects on rat offspring after prenatal exposure to ethanol. Neurotoxicol. Teratol..

[140] Osborn J.A., Kim C.K., Steiger J., Weinberg J. (1998). Prenatal ethanol exposure differentially alters behavior in males and females on the elevated plus maze. Alcohol. Clin. Exp. Res..

[141] Osborn J.A., Yu C., Gabriel K., Weinberg J. (1998). Fetal ethanol effects on benzodiazepine sensitivity measured by behavior on the elevated plus-maze. Pharmacol. Biochem. Behav..

[142] Wieczorek L., Fish E.W., O’Leary-Moore S.K., Parnell S.E., Sulik K.K. (2015). Hypothalamic-pituitary-adrenal axis and behavioral dysfunction following early binge-like prenatal alcohol exposure in mice. Alcohol.

[143] Popova N.K., Morozova M.V., Naumenko V.S. (2011). Ameliorative effect of BDNF on prenatal ethanol and stress exposure-induced behavioral disorders. Neurosci. Lett..

[144] Baculis B.C., Diaz M.R., Valenzuela C.F. (2015). Third trimester-equivalent ethanol exposure increases anxiety-like behavior and glutamatergic transmission in the basolateral amygdala. Pharmacol. Biochem. Behav..

[145] Diaz M.R., Jotty K., Locke J.L., Jones S.R., Valenzuela C.F. (2014). Moderate Alcohol Exposure during the Rat Equivalent to the Third Trimester of Human Pregnancy Alters Regulation of GABAA Receptor-Mediated Synaptic Transmission by Dopamine in the Basolateral Amygdala. Front. Pediatr..

[146] Patten A.R., Brocardo P.S., Sakiyama C., Wortman R.C., Noonan A., Gil-Mohapel J., Christie B.R. (2013). Impairments in hippocampal synaptic plasticity following prenatal ethanol exposure are dependent on glutathione levels. Hippocampus.

[147] Puglia M.P., Valenzuela C.F. (2010). Repeated third trimester-equivalent ethanol exposure inhibits long-term potentiation in the hippocampal CA1 region of neonatal rats. Alcohol.

[148] Subbanna S., Shivakumar M., Psychoyos D., Xie S., Basavarajappa B.S. (2013). Anandamide-CB1 Receptor Signaling Contributes to Postnatal Ethanol-Induced Neonatal Neurodegeneration, Adult Synaptic and Memory Deficits. J. Neuoscience.

[149] Patten A.R., Fontaine C.J., Christie B.R. (2014). A comparison of the different animal models of fetal alcohol spectrum disorders and their use in studying complex behaviors. Front. Pediatr..

[150] Richardson D.P., Byrnes M.L., Brien J.F., Reynolds J.N., Dringenberg H.C. (2002). Impaired acquisition in the water maze and hippocampal long-term potentiation after chronic prenatal ethanol exposure in the guinea-pig. Eur. J. Neurosci..

[151] Fontaine C.J., Pinar C., Yang W., Pang A.F., Suesser K.E., Choi J.S.J., Christie B.R. (2019). Impaired Bidirectional Synaptic Plasticity in Juvenile Offspring Following Prenatal Ethanol Exposure. Alcohol. Clin. Exp. Res..

[152] Kervern M., de Ferron B.S., Alaux-Cantin S., Fedorenko O., Antol J., Naassila M., Pierrefiche O. (2015). Aberrant NMDA-dependent LTD after perinatal ethanol exposure in young adult rat hippocampus. Hippocampus.

[153] Honse Y., Nixon K.M., Browning M.D., Leslie S.W. (2003). Cell surface expression of NR1 splice variants and NR2 subunits is modified by prenatal ethanol exposure. Neuroscience.

[154] Nixon K., Hughes P.D., Amsel A., Leslie S.W. (2002). NMDA receptor subunit expression following early postnatal exposure to ethanol. Brain Res. Dev. Brain Res..

[155] Nixon K., Hughes P.D., Amsel A., Leslie S.W. (2004). NMDA receptor subunit expression after combined prenatal and postnatal exposure to ethanol. Alcohol. Clin. Exp. Res..

[156] Samudio-Ruiz S.L., Allan A.M., Sheema S., Caldwell K.K. (2010). Hippocampal N-methyl-D-aspartate receptor subunit expression profiles in a mouse model of prenatal alcohol exposure. Alcohol. Clin. Exp. Res..

[157] Martin D., Savage D.D., Swartzwelder H.S. (1992). Effects of prenatal ethanol exposure on hippocampal ionotropic-quisqualate and kainate receptors. Alcohol. Clin. Exp. Res..

[158] Montagud-Romero S., Cantacorps L., Fernandez-Gomez F.J., Nunez C., Minarro J., Rodriguez-Arias M., Milanes M.V., Valverde O. (2021). Unraveling the molecular mechanisms involved in alcohol intake and withdrawal in adolescent mice exposed to alcohol during early life stages. Prog. Neuro Psychopharmacol. Biol. Psychiatry.

[159] Vaglenova J., Pandiella N., Wijayawardhane N., Vaithianathan T., Birru S., Breese C., Suppiramaniam V., Randal C. (2008). Aniracetam reversed learning and memory deficits following prenatal ethanol exposure by modulating functions of synaptic AMPA receptors. Neuropsychopharmacology.

[160] Wijayawardhane N., Shonesy B.C., Vaithianathan T., Pandiella N., Vaglenova J., Breese C.R., Dityatev A., Suppiramaniam V. (2008). Ameliorating effects of preadolescent aniracetam treatment on prenatal ethanol-induced impairment in AMPA receptor activity. Neurobiol. Dis..

[161] Anggono V., Huganir R.L. (2012). Regulation of AMPA receptor trafficking and synaptic plasticity. Curr. Opin. Neurobiol..

[162] Paoletti P., Bellone C., Zhou Q. (2013). NMDA receptor subunit diversity: Impact on receptor properties, synaptic plasticity and disease. Nat. Rev. Neurosci..

[163] Lv X.F., Sun L.L., Cui C.L., Han J.S. (2015). NAc Shell Arc/Arg3.1 Protein Mediates Reconsolidation of Morphine CPP by Increased GluR1 Cell Surface Expression: Activation of ERK-Coupled CREB is Required. Int. J. Neuro Psychopharmacol..

[164] Liu S.J., Zukin R.S. (2007). Ca2+-permeable AMPA receptors in synaptic plasticity and neuronal death. Trends. Neurosci..

[165] Wang X., Carlson V.C.C., Studholme C., Newman N., Ford M.M., Grant K.A., Kroenke C.D. (2020). In Utero MRI identifies consequences of early-gestation alcohol drinking on fetal brain development in rhesus macaques. Proc. Natl. Acad Sci. USA.

[166] Uchida S., Teubner B.J., Hevi C., Hara K., Kobayashi A., Dave R.M., Shintaku T., Jaikhan P., Yamagata H., Suzuki T. (2017). CRTC1 Nuclear Translocation Following Learning Modulates Memory Strength via Exchange of Chromatin Remodeling Complexes on the Fgf1 Gene. Cell Rep..

[167] Manji S., Pei J., Loomes C., Rasmussen C. (2009). A review of the verbal and visual memory impairments in children with foetal alcohol spectrum disorders. Dev. Neurorehabil..

[168] Rasmussen C., Andrew G., Zwaigenbaum L., Tough S. (2008). Neurobehavioural outcomes of children with fetal alcohol spectrum disorders: A Canadian perspective. Paediatr. Child Health.

[169] Rasmussen C., Horne K., Witol A. (2006). Neurobehavioral functioning in children with fetal alcohol spectrum disorder. Child Neuropsychol..

